# Oxygenator oxygen transfer performance during cardiopulmonary bypass of the Sorin^®^ Inspire 6F^©^

**DOI:** 10.1177/02676591261438976

**Published:** 2026-03-24

**Authors:** David Max Riesterer, Christoph Eisner, Jacob Wojdyla, Jan Turra

**Affiliations:** 1 Department of Cardiac Surgery, 27178 University Hospital Heidelberg; 2 Department of Anesthesiology, 27178 University Hospital Heidelberg; 3 Department of Cardiothoracic Surgery, 23536 Kaiser Sunnyside Medical Center

**Keywords:** cardiopulmonary bypass, Inspire 6F, oxygen transfer function, oxygen transfer volume, oxygenator, VO_2_

## Abstract

**Background:**

Oxygenator oxygen transfer performance is typically measured by manufacturers under standardized, static laboratory conditions. However, these parameters do not adequately reflect the dynamic environment of clinical cardiopulmonary bypass (CPB). To align with modern goal-directed perfusion standards - specifically the maintenance of physiological PaO2 levels to reduce oxidative stress - perfusionists require a reliable tool to titrate FiO2 based on real-time oxygen demand. This study aimed to develop a clinical oxygen transfer function for the Sorin® Inspire 6F© oxygenator.

**Methods:**

We retrospectively analyzed 420 blood gas samples and hemodynamic parameters from 54 adult patients undergoing CPB with the Sorin® Inspire 6F©. Oxygen transfer (VO2) was calculated via the Fick principle. Using the methodology established by Hamilton et al., a validation subset (*n* = 57) of samples within a target PaO2 range of 150 ± 5 mmHg was used to derive a specific correlation function between FiO2 and VO2 via linear regression analysis.

**Results:**

A significant correlation was observed between FiO2 and oxygen transfer. For the validation subset, the derived oxygen transfer function for the Sorin® Inspire 6F© was VO_2_ = 284.34 x FiO2 (R^2^ = 0.6185). In contrast, the correlation for the entire clinical dataset (*n* = 420) yielded an R^2^ of 0.4583. Comparison with existing literature indicates that the Inspire 6F© has a lower transfer capacity compared to larger membrane surface area oxygenators, as expected.

**Conclusions:**

The newly developed oxygen transfer function provides a practical, evidence-based tool for the clinical setting. It allows perfusionists to adjust the FiO2 accurately to meet patient-specific oxygen requirements and maintain desired PaO2 levels. Implementing this function in routine practice can enhance standardization and improve patient safety by preventing hyperoxygenation.

## Background

Standardized testing of oxygenator performance is typically conducted according to the guidelines of the Association for the Advancement of Medical Instrumentation and the International Organization for Standardization (AAMI/ISO 7199).^1^ While these protocols are designed to simulate physiological conditions, they rely on fixed parameters - such as a hemoglobin concentration of 12 g/dl, a venous oxygen saturation (S_v_O_2_) of 70%, and a fraction of inspired oxygen (F_i_O_2_) of 1.0. However, these static laboratory conditions fail to account for the dynamic nature of cardiopulmonary bypass (CPB) or the diverse physiological variables encountered in clinical practice. As early as 1996, Seger et al. observed that standardized bench testing is limited in its ability to reflect complex clinical situations.^2^ Despite these concerns, significant progress in quantifying real - time oxygenator performance was not documented until Hamilton et al. (2017).^3^ They introduced a novel methodology to assess oxygen transfer (VO_2_) during active CPB, allowing perfusionists to titrate F_i_O_2_ precisely to achieve a target arterial oxygen partial pressure (P_a_O_2_) of 150 mmHg based on patient-specific oxygen demand. While Hamilton et al. validated this approach using the Medtronic^®^ Fusion oxygenator, the methodology is theoretically applicable to any hollow-fiber oxygenator. One such widely utilized system is the Sorin^®^ Inspire 6F^©^ (LivaNova^®^, London, UK). To date, however, clinical performance data for the Inspire 6F^©^ remain unavailable. Current literature emphasizes that modern perfusion practice should prioritize the reduction of oxidative stress by avoiding hyperoxygenation.^4^ Excessively high P_a_O_2_ levels above physiological ranges facilitate the formation of oxygen radicals, which can be mitigated through precise F_i_O_2_ management. To move beyond the “trial and error” approach still common in many clinical settings and to improve patient safety, this study analyzed 420 blood gas samples from 54 patients. Our objective was to establish a specific oxygen transfer correlation function for the Sorin^®^ Inspire 6F^©^, providing the perfusion community with a reliable tool for accurate F_i_O_2_ titration.

## Methods

### Study design and population

The objective of this retrospective study was to derive a specific oxygen transfer function correlating VO_2_ and F_i_O_2_ for the Sorin^®^ Inspire 6F^©^ oxygenator using routine clinical data. Following approval by the Ethics Committee of the Medical Faculty of Heidelberg (S-795/2021), the study included all adult patients with a hemoglobin concentration >6 g/dl who underwent cardiopulmonary bypass (CPB) using the Inspire 6F^©^ system between April 1st and October 31st.

### Extracorporeal circuit and data acquisition

In all cases, an open-system reservoir (Sorin^®^ Inspire 6F^©^) was utilized in conjunction with a Stöckert S5 heart-lung machine (LivaNova^®^, Munich, Germany). The circuit featured an integrated arterial filter with a total oxygenator membrane surface area of 1.4 m^2^ and a rated maximum blood flow of 6 l/min. Continuous data logging and blood flow monitoring were performed using the Data Management System (DMS; Stöckert, Munich, Germany). To ensure high-quality data, blood gas samples were only collected during periods of hemodynamic steady-state, defined as a minimum of 2 min without adjustments to CPB parameters. Sampling was specifically avoided during volume substitution, the administration of cardioplegia, or periods of mechanical ventilation.

### Laboratory analysis and calculations

Simultaneous arterial and venous blood gas samples were analyzed using the Siemens RapidPoint 4000 system (Siemens^®^ Healthcare GmbH, Erlangen, Germany). Measurements were performed using the pH-stat strategy and were temperature-corrected within a range of 34°C to 37°C. Throughout the study, the fresh gas inlet flow was maintained at a constant rate between 1.5 l/min and 2.0 l/min. VO_2_ was calculated according to the Fick principle. To account for varying oxygenation levels, a corrected F_i_O_2_ (cF_i_O_2_:150 mmHg) - representing the F_i_O_2_ required to achieve a target P_a_O_2_ of 150 mmHg - was derived using the methodology proposed by Hamilton et al.^3^ (see Appendix I for detailed calculations).

### Statistical analysis

Data processing, descriptive statistics (expressed as mean ± standard deviation), and linear regression analyses were performed using Microsoft Excel^®^ (Microsoft Corp.^©^, Redmond, WA, USA). The regression model utilized the least-squares method to determine the specific transfer characteristics of the Inspire 6F^©^ oxygenator.

### Statistical modeling of oxygen transfer performance

To characterize the gas transfer efficiency of the Inspire 6F oxygenator, a mathematical model was developed to define the relationship between oxygen consumption and the blender setting. Given that oxygenator performance is primarily a function of membrane efficiency, a linear regression model was utilized to correlate calculated VO_2_ with the cF_i_O_2_:150 mmHg.

For each data point, VO_2_ was calculated using the Fick principle, derived from the pump flow and the arterio-venous oxygen content difference. To ensure comparability across varying oxygenation levels, the measured F_i_O_2_ was normalized to a target P_a_O_2_ of 150 mmHg cF_i_O_2_:150 mmHg. This correction was performed according to the methodology established by Hamilton et al.,^3^ adapted specifically for the performance characteristics of the Inspire 6F.

The oxygen transfer function was derived via simple linear regression using the least-squares method (y = ax + b). In this model, the slope (a) represents the specific oxygen transfer efficiency of the Inspire 6F, while the *y*-intercept (b) accounts for potential systemic offsets.

To ensure model integrity, regression parameters were calculated for both the entire dataset and a targeted validation subset, the latter consisting only of samples where the actual measured P_a_O_2_ was within 150 ± 5 mmHg. Model fit and precision were quantified using the coefficient of determination (R^2^), and the statistical significance of the regression coefficients was evaluated.

Linear regression analyses were performed with the intercept fixed at the origin (b = 0), as a zero-driving pressure or concentration must physiologically result in zero oxygen transfer; supplementary evaluation with a floating intercept confirmed that the calculated offsets were negligible and did not significantly affect the regression slopes.

## Results

A total of 420 arterial and venous blood gas samples of 54 patients were taken. Table 1 shows the CPB parameters that were recorded.

**Table 1.** Clinical and perfusion parameters during CPB. This table presents the demographic and intraoperative data for the study population (*n* = 54 patients). Values are expressed as Mean ± Standard Deviation (SD) and range (minimum–maximum). The data reflect the steady-state conditions during which the 420 blood gas samples were collected.

	Mean ± SD	Range (Min-Max)
FiO_2_ (%)	0.54 ± 0.11	0.31–1
Blood flow (l/min)	5.16 ± 0.6	2.72–6.42
paO_2_ (mmHg)	180.7 ± 77	60–529
pvO_2_ (mmHg)	45.2 ± 6.4	31.4–64.4
SaO_2_ (%)	99 ± 1	89.2–99.8
SvO_2_ (%)	78 ± 6	59.2–97.6
cHb (g/dl)	9.14 ± 1.27	5.6–12.8
BSA (m^2^)	2.07 ± 0.24	1.08–2.56

Table 2 shows the calculated oxygen concertation data of the venous and arterial blood gas sample sets as well as the oxygen transfer rate.

**Table 2.** Summary of blood gas analysis and calculated oxygen transfer data. The table summarizes the oxygenation parameters derived from the 420 arterial and venous blood gas samples. It includes measured partial pressures, hemoglobin concentrations, and the resulting VO_2_ calculated via the Fick principle. Values are presented as Mean ± SD.

	Mean ± SD	Range (Min-Max)
caO_2_ (ml/dl)	12.62 ± 1.68	7.94–17.86
cvO_2_ (ml/dl)	9.66 ± 1.65	5.36–16.79
VO_2_ (ml)	153 ± 39.9	32–277.9
cFiO_2_ :150 mmHg	0.5 ± 0.11	0.195–0.821

57 of the 420 blood gas samples that were taken were within the desired PaO_2_ range of 145 mmHg and 155 mmHg. These samples were taken to develop the FiO_2_ to VO_2_ function that is shown in Figure 1. The regression analysis of the plotted function showed an R^2^ value of 0.6185. The proposed function in this paper to determine the required FiO_2_ to transfer the desired volume of oxygen (VO_2_) with the Sorin^®^ Inspire 6F^©^ oxygenator was VO_2_ = 284.34 * FiO_2_.

**Figure 1.**
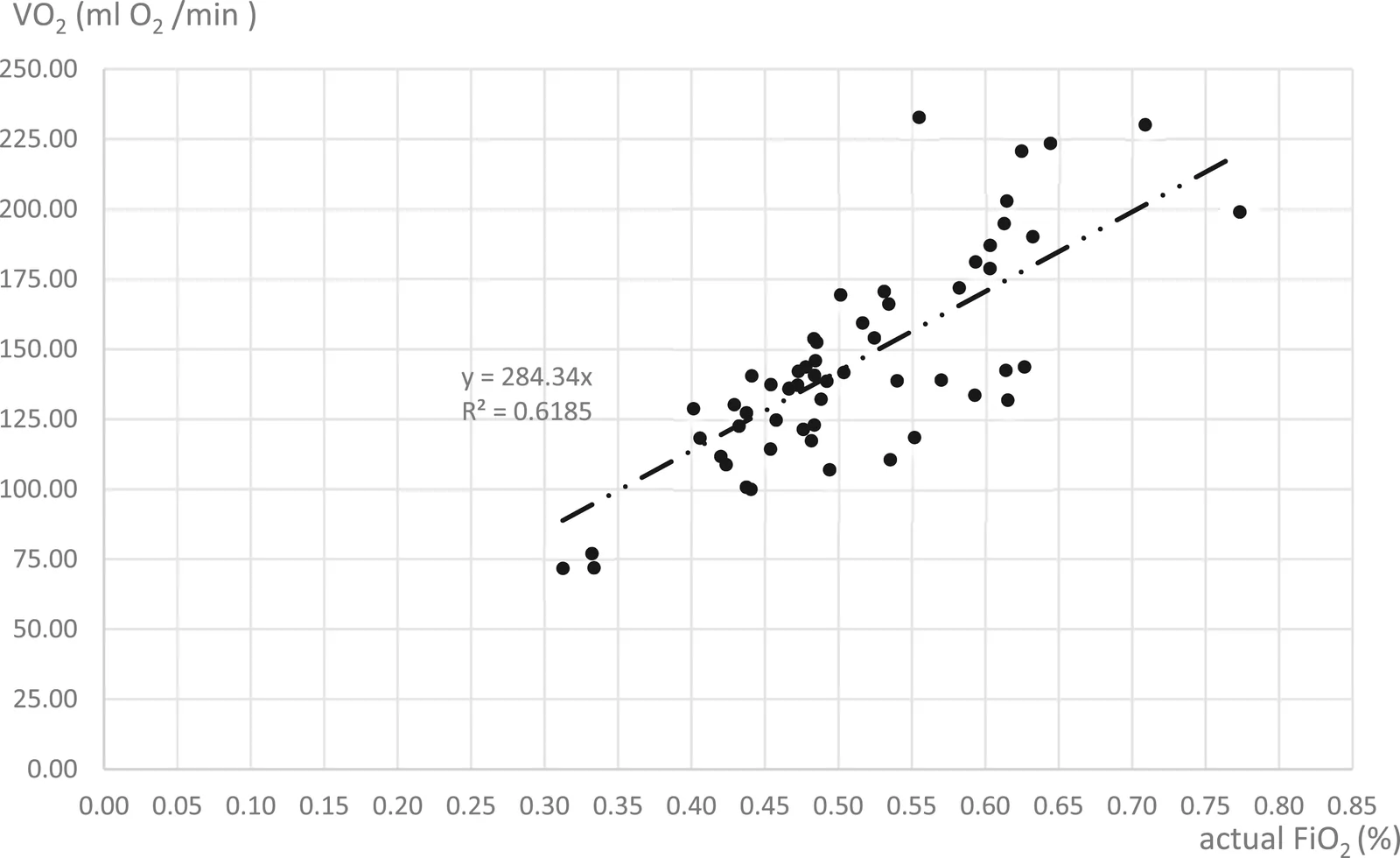
Primary oxygen transfer function for the Sorin^®^ Inspire 6F^©^. This scatter plot illustrates the linear relationship between the actual fraction of F_i_O_2_ and the calculated VO_2_ for the validation subset (*n* = 57). This subset exclusively includes blood gas samples where the target P_a_O_2_ was successfully maintained within the physiological range of 150 ± 5 mmHg. The regression line (VO_2_ = 284.34 x F_i_O_2_; R^2^ = 0.6185) serves as the predictive model for clinical F_i_O_2_ titration.

The regression function of all documented FiO_2_ values to the calculated and correlating oxygen transfer volume is shown in Figure 2 where the R_2_ value was 0.4583.

**Figure 2.**
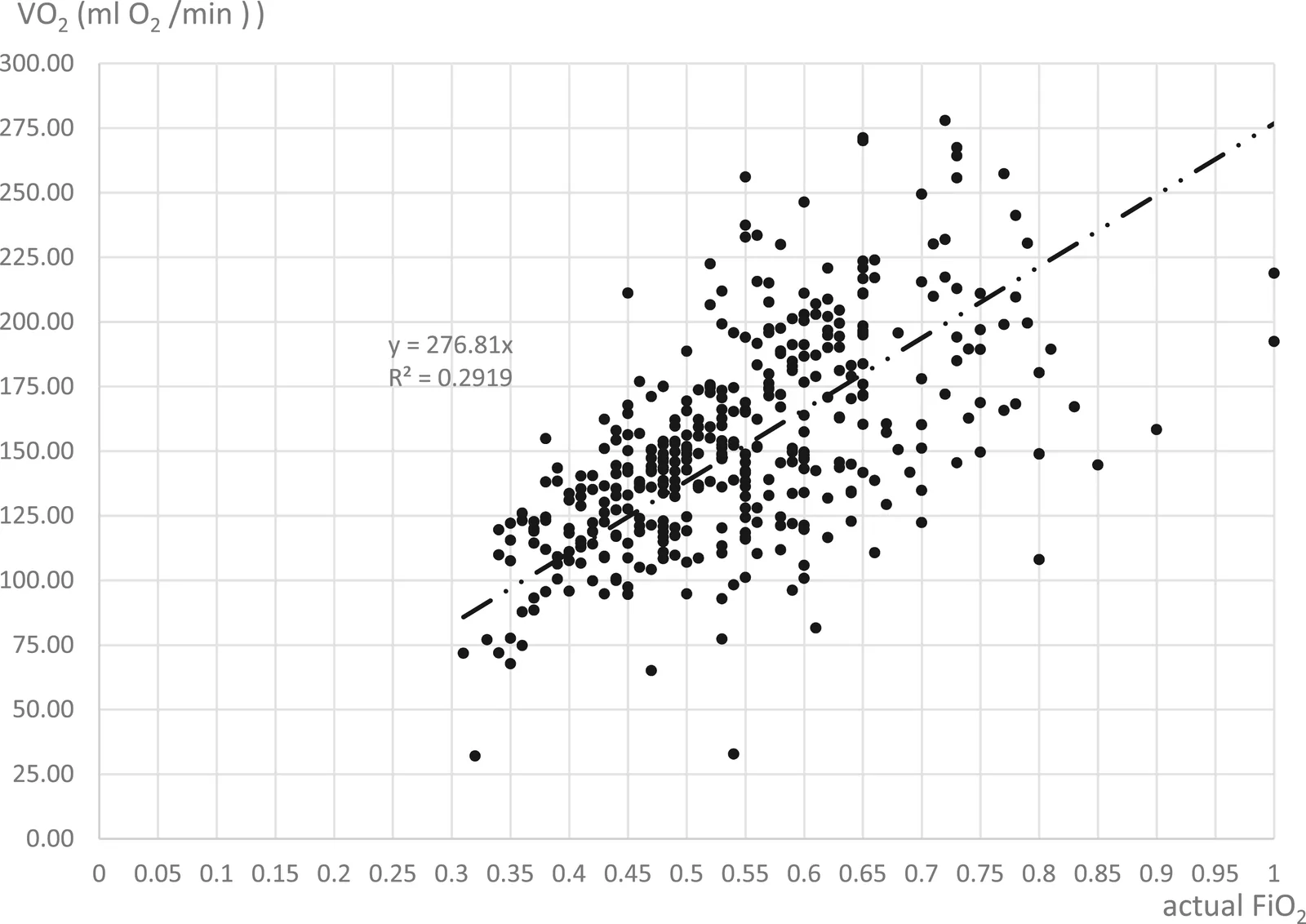
Correlation of actual F_i_O_2_ and oxygen transfer for the total dataset. The graph shows the regression analysis of all documented F_i_O_2_ values against the calculated VO_2_ for the complete dataset (*n* = 420 samples). Unlike Figure 1, this plot includes all measurements regardless of whether the target P_a_O_2_ was reached. The lower coefficient of determination (R^2^ = 0.4583) reflects the clinical variability and the impact of P_a_O_2_ deviations on the oxygen transfer relationship.

The corrected FiO_2_ value to 150 mmHg to the oxygen transfer volume is shown in Figure 3. The regression analysis showed an R^2^ value of 0.2919.

**Figure 3.**
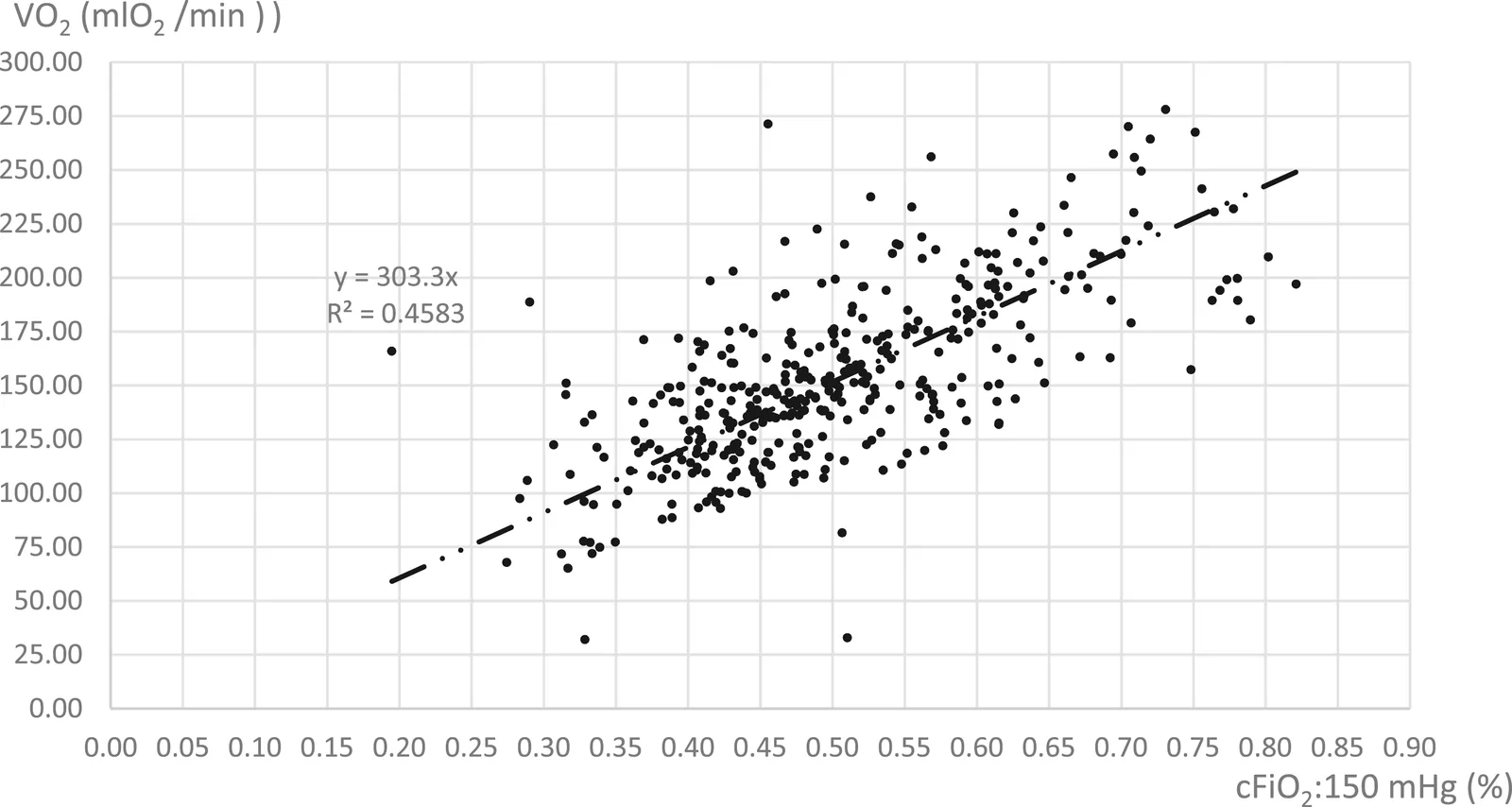
Relationship between cF_i_O_2_ and oxygen transfer. This plot displays the oxygen transfer volume VO_2_ in relation to the cF_i_O_2_, which was mathematically normalized to a target P_a_O_2_ of 150 mmHg for all 420 samples. The regression analysis shows an R^2^ value of 0.2919. This representation highlights the challenges of predicting transfer performance across a broad range of non-steady-state clinical conditions.

## Discussion

In this study, we established a clinical oxygen transfer function for the Sorin^®^ Inspire 6F^©^ oxygenator: VO_2_ = 284.34 x FiO_2_. This formula provides perfusionists with a practical tool to titrate FiO_2_ according to patient-specific metabolic requirements, aiming to maintain physiological PaO_2_ levels and mitigate the risks associated with hyperoxia during CPB. Manufacturer-provided data often offer only a baseline estimation of gas exchange capabilities, typically derived under standardized AAMI/ISO conditions using an FiO_2_ of 1.0 and fixed hematologic parameters. As shown in Figure 4, these performance curves primarily correlate oxygen transfer with blood flow.^5^ However, such data do not account for the variable FiO_2_ settings used clinically to manage oxygenation. By adopting the methodology established by Hamilton et al.,^3^ which utilizes the Fick principle and FiO_2_ normalization to a target PaO_2_ of 150 mmHg, we have bridged the gap between bench testing and real-world clinical application for the Inspire 6F^©^.

**Figure 4.**
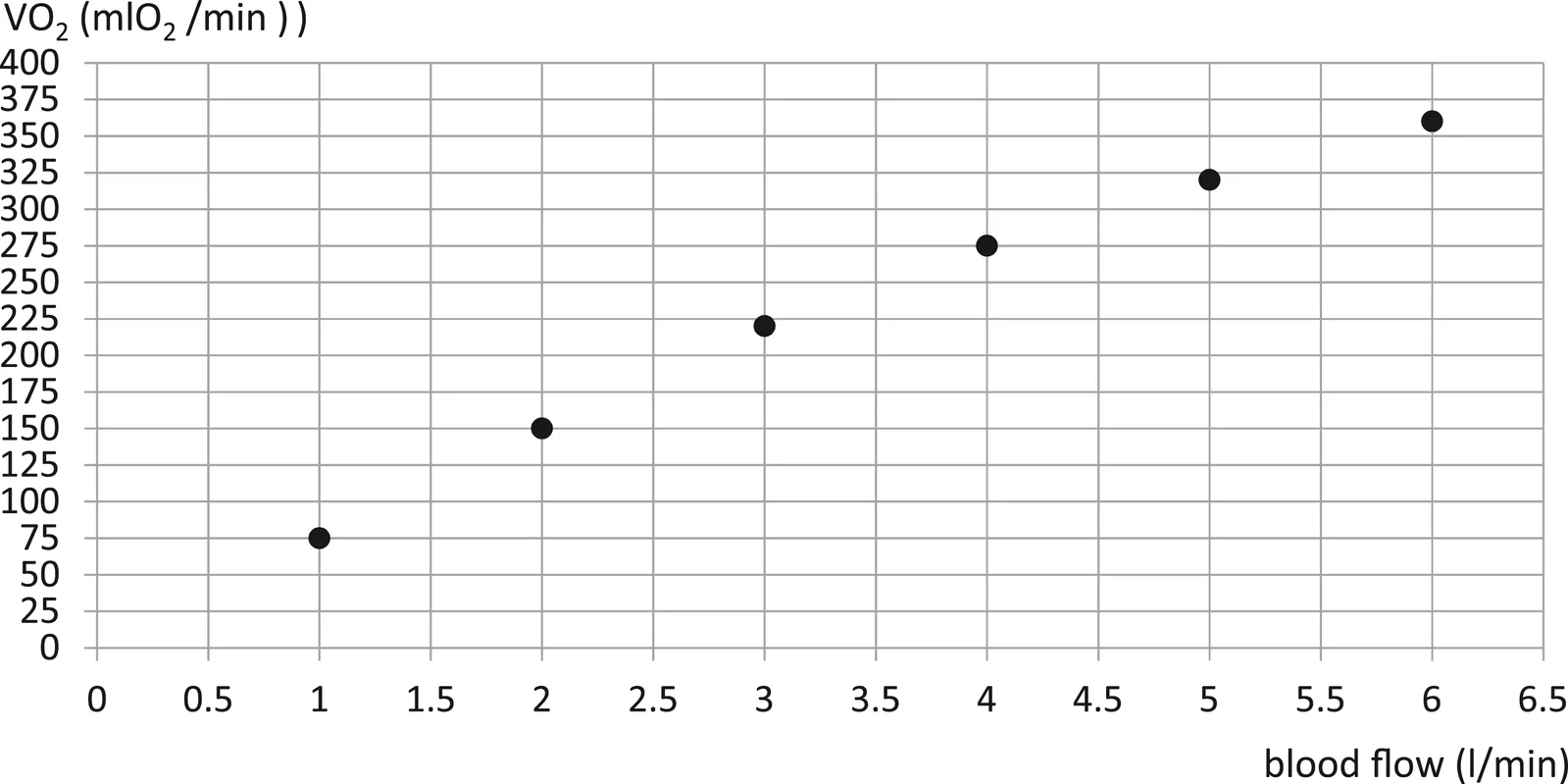
Correlation between blood flow and VO_2_ for the Sorin^®^ Inspire 6F^©^.

Comparing our results with previous data, Hamilton et al. reported a trend line of VO_2_ = 462.03 x FiO_2_ - 43.025 for the Medtronic^®^ Fusion oxygenator.^3^ Our findings indicate that the Medtronic Fusion facilitates approximately 38.5% higher oxygen transfer than the Sorin^®^ Inspire 6F^©^ at identical FiO_2_ settings. This discrepancy is primarily attributable to the difference in membrane surface area; the Inspire 6F^©^ (1.4 m^2^) is significantly smaller than the Fusion (2.5 m^2^), resulting in a reduced diffusion interface for hemoglobin loading.

Beyond surface area, the diffusion capacity of artificial lungs is governed by the contact time between blood and the fiber bundle, the partial pressure gradient between the gas and blood phases, and the oxygenator shunt fraction.^6,7^ The shunt fraction - the proportion of blood flow that bypasses effective gas exchange - is a critical but often understudied factor in clinical settings.^6^ While we have not specifically quantified the shunt fraction for the Inspire 6F^©^, it likely contributes to the observed transfer characteristics.

The calculated oxygen transfer function demonstrated a moderate to strong correlation (R^2^ = 0.6185), confirming that FiO_2_ management is the primary lever for controlling arterial oxygenation. However, several clinical limitations must be acknowledged. CPB is a highly dynamic system; variables such as the return of unoxygenated blood via vent and suction pumps, fluctuations in S_v_O_2_, and systemic inflammatory responses can influence oxygen transfer efficiency.^3^ Although we ensured a steady-state for sampling by avoiding periods of cardioplegia administration and volume substitution, real-time clinical conditions remain inherently variable.

Consequently, the proposed formula VO_2_ = 284.34 x FiO_2_ should be regarded as a robust clinical approximation rather than an absolute physical constant. Nevertheless, it provides a valuable framework for transitioning from empirical “trial and error” FiO_2_ adjustments to a more standardized, evidence-based approach in the operation of the Sorin^®^ Inspire 6F^©^.

## Conclusion

In light of the limited manufacturer data regarding the oxygen transfer performance of the Sorin^®^ Inspire 6F^©^ under variable F_i_O_2_ settings, this study established a clinical correlation function derived from real-world bypass data. The derived formula, VO_2_ = 284.34 x F_i_O_2_, serves as a practical bedside tool, enabling perfusionists to titrate oxygen delivery more precisely to meet individual patient metabolic demands while maintaining physiological PaO_2_ levels. By providing performance data that reflect the complexities of daily clinical practice rather than idealized laboratory conditions, this study supports the transition toward goal-directed perfusion. Implementing such standardized F_i_O_2_ titration protocols can help mitigate the risks of hyperoxygenation-induced oxidative stress, ultimately promoting higher standards of practice and enhancing patient safety during cardiopulmonary bypass.

## Appendix I

Fick equation; where VO_2_ is referred to as oxygen transfer volume:

$$VO_{2}=Q*10*\left(c_{a}O_{2}-c_{v}O_{2}\right)$$

where Q is the perfusion flow in ml/min and caO_2_ as well as c_v_O_2_ are described as follow:

$$c_{a}O_{2}=Hb*S_{a}*HFZ+p_{a}O_{2}*k$$

$$c_{v}O_{2}=Hb*S_{v}*HFZ+p_{v}O_{2}*k$$

where Hb is the haemoglobin concertation, S_a/v_ is the oxygen saturation of haemoglobin in arterial and venous blood in decimal form, p_a/v_O_2_ are the partial pressures of oxygen of arterial and venous blood in mmHg, k is a factor that describes the amount of oxygen that is dissolved in the blood and the factor HFZ is the Huefner factor in ml O_2_/g Hb.

Using the proposed equation from Hamilton et al., the F_i_O_2_ was calculated to the cF_i_O_2_:150 mmHg if the P_a_O_2_ was not within the physiological range of 150 mmHg, in order to calculate the F_i_O_2_ that would be needed to achieve a P_a_O_2_ of 150 mmHg.^2^ The equation of Hamilton et al. was used as follow^3^:

$$cFiO_{2}:150\;mmHg=\left(FiO_{2}-\left(\frac{p_{a}O_{2}}{p_{b}-p_{H2O}}\right)\right)+0.21$$

where F_i_O_2_ is the oxygen fraction of the inlet fresh gas into the oxygenator that was used, P_a_O_2_ is the oxygen partial pressure of arterial blood that was measured corresponding to the used F_i_O_2_, p_b_ is the barometric pressure of 713 mmHg in Heidelberg, p_H2O_ is the water vapor pressure of 47 mmHg and 0.21 is the oxygen concentration in the atmospheric gas.
